# Shifts in Soil Bacterial Community Composition of Jujube Orchard Influenced by Organic Fertilizer Amendment

**DOI:** 10.4014/jmb.2406.06037

**Published:** 2024-10-30

**Authors:** Heesoon Park, Kiyoon Kim, Denver I. Walitang, Riyaz Sayyed, Tongmin Sa

**Affiliations:** 1 Department of Environmental and Biological Chemistry, Chungbuk National University, Cheongju 28644, Republic of Korea; 2 Jujube Research Institute, Chungcheongbuk-do Agricultural Research and Extension Services, Boeun, Chungbuk 28902, Republic of Korea; 3 National Forest Seed Variety Center, Chungju 27495, Republic of Korea; 4 College of Agriculture, Forestry and Environmental Science, Romblon State University, Romblon 5505, Philippines; 5 Department of Biological Sciences and Chemistry, University of Nizwa, Nizwa 616, Sultanate of Oman; 6 The Korean Academy of Science and Technology, Seongnam 13630, Republic of Korea

**Keywords:** Jujube, soil chemical properties, bacterial community, oil cake, organic fertilization, Illumina sequencing

## Abstract

Organic fertilizer application in agricultural land is known to improve soil microbial processes, fertility, and yield. In particular, the changes in soil chemical composition due to multi-year application of organic fertilizers are thought to alter the microbial community. Here, the effects of organic fertilization with oil-cake amendments (OC) on soil bacterial diversity, community profile, and enzyme activity were evaluated and compared to those amended with chemical fertilizer (NPK). Diversity indices show that the application of organic fertilizer potentially increases microbial diversity as well as the number of different microbial groups. The ordination plot distinguished and clustered both treatments, showing the differential effects of soil chemical factors on the microbial communities in each treatment. *Proteobacteria*, Verrucomicrobia, and Bacteriodetes were significantly more abundant in OC-amended soil than in the NPK soil, indicating alterations in community structure, composition, and diversity, concurrent to the changes in the pH, Ca, and Mg contents of the soil. These shifts in bacterial community structure and composition, partially explained by differences in soil chemical factors, could be observed from the phylum to the genus level in NPK- and OC-amended soils. The OC soil contained a significantly higher abundance of predicted genes corresponding to enzymes related to biogeochemical cycling, decomposition, and plant growth promotion. Collectively, these results support the use of an unconventional organic fertilizer positively altering bacterial populations in jujube orchards. The application of an unconventional organic fertilizer improved microbial diversity and enhanced ecosystem functions related to biogeochemical cycles, mineralization, and plant growth promotion.

## Introduction

Jujubes are the most important fruit-producing *Ziziphus* species in the *Rhamnaceae* family [1]. They are rich in various secondary metabolites associated with nutrition and biological activities [2]. Free amino acids, phenolics, antioxidant activities and flavonoids from jujube have health-promoting effects that vary and may depend on the cultivar and growing conditions [3]. Jujubes are cultivated using various methods including conventional orchards, intensive orchards, intercropping, and greenhouse cultivation in which farmyard manure and other types of fertilization have typically been applied [4]. Most of the current jujube research focuses on germplasm, variety and breeding improvement, propagation systems, cultivation systems, pest and disease management, and postharvest studies [5]. Although fertilizer application and management are not the main priority in jujube research, it is an essential component for eco-friendly, high-quality food safety.

In most cases, application of organic fertilizers in agroecosystems usually entails utilization of manure, plant residues, or combinations of both, either fresh or composted [6]. Also, the adoption of organic management systems should incorporate not just their perceived beneficial effects, but also their potential contribution to global environmental issues like greenhouse gas emissions, as exemplified in rice agroecosystems [7]. To evaluate soil quality, we need to also include biological and biochemical components of the soil as essential indicators of sustainable agriculture with the goal of mitigating environmental issues [8, 9]. This is in stark contrast with conventional evaluation of soil quality based mainly on crop productivity and economic value. The application of organic fertilizers, especially with long-term use, can bring about changes in the physicochemical components of the soil, thereby altering the microbial components and leading to changes that distinctly separate soils with and without fertilizer application. These changes ultimately impact the biochemical features of soils, affecting their overall function [10, 11].

In Korea, organic fertilizer-based research is promoted for eco-friendly agriculture while oil cake is an unconventional organic fertilizer used widely in Korean agriculture, including in jujube orchards [12]. Oil cake is easily mineralized by soil microbes and the nutrients are utilized by crops, improving growth and productivity [13, 14]. Oil cake has 1~3 times higher nitrogen content compared to livestock compost, and its nitrogen is mainly organic in nature [15].

Furthermore, microbiomes are affected by various factors including fertilization [16], irrigation [17], and tillage [18]. On the other hand, soil microbial enzymes play critical roles in agroecosystems, catalyzing important reactions necessary for nutrient cycling and decomposition, which in turn supports various biological processes important for soil microorganisms [19]. Previous studies have focused on unraveling the soil microbial community profiles in different agricultural fields treated with conventional organic fertilizers [20-24]. The application of organic fertilizer could alter soil properties [10, 11, 15], soil microbial communities and function [20-23], and even crop yield and quality [24]. Although there are numerous studies on the effect of organic fertilization on agroecosystems, few studies have focused on the effect of oil cake-based fertilization on the communities of microbes in jujube orchards.

Hence, in this study, we characterized the bacterial community composition of jujube orchards treated with oil cake-based organic fertilizer and compared them with conventional NPK fertilized treatments. This work underscores the dynamic changes and shifts in bacterial community structure, composition, and diversity in jujube orchard due to the application of an unconventional oil cake-based organic fertilizer compared to those applied with conventional chemical fertilizers. In addition, this study correlates how subsequent changes in soil chemical parameters due to different fertilizer application are attributed to the observed bacterial community shifts. To gain insight into the potential functional relevance of the observed microbial communities, the computational abundances of gene families associated with common soil enzymes, decomposition-related, and plant growth promotion-related enzyme-encoding genes were also predicted.

## Materials and Methods

### Research Sites, Fertilizer Application and Sampling

The Jujube Research Institute, located in Boeun, South Korea (36°34'39.6"N 127°44'54.1"E), allocated the study sites for this research. The jujube variety used in this study was Bokjo (*Ziziphus jujuba* var. inermis (Bunge, Rehder), and the distance between trees was maintained at 4 m × 2 m. The fertilizer treatments were: (1) NPK: chemical fertilizer and (2) OC: organic fertilizer mainly composed of oil cake. The fertilization was carried out according to the nitrogen content of the soil, and irrigation was performed periodically. Urea was used as the nitrogen fertilizer, fused phosphate was used as phosphate fertilizer, potassium sulfate was used to provide potassium, and oil cake was used as the organic fertilizer. The oil cake-based organic fertilizer contained a mixed ratio of 30% rape seed cake, 58% castor oil cake, and 12% rice bran cake. The jujube orchards were maintained and treated with the same fertilizer amendments for 4 years. Topsoil (0~30 cm deep) samples were collected from the plastic rain shelter in August 2020. Individual samples from three trees were pooled together and considered as one composite sample. Each treatment has three composite replicates. Roots and stones were removed and the soils were sieved (2.0-mm mesh). Samples were packed on ice for laboratory transport to conduct soil chemical analyses and DNA isolation. Soil samples were separated into two portions, with one portion being directly used for DNA isolation, and the other for soil chemical analyses. The samples for chemical analyses were air-dried under shade following standard protocols [25]. The soil chemical analyses included soil pH, total N, electrical conductivity (EC), NO_3_-N, NH_4_-N, soil organic matter content (OM), available P_2_O_5_, and soil K, Ca, and Mg contents. All soil chemical testing procedures were done according to standard methodologies established by NIAST [25].

### Extraction of Soil DNA

The soil DNA extraction kit developed by QIAGEN (DNeasy PowerSoil Pro Kit; Germany) was used for isolating DNA. A NanoDrop spectrophotometer and fluorometer (Thermo Fisher; Qubit, Life Technologies Co., USA) were used to check DNA quality and quantity. Final samples were always stored at −80°C.

### DNA Sequencing and Analyses

Sequencing preparation and sequencing were conducted at Nexbio (Republic of Korea) followed the Illumina 16S Metagenomics sequencing library protocols through the Illumina HiSeq 2000 DNA sequencer. For the library, primers targeting the V3-V4 region PCR were used with sequences 341F (5'-ACTCCTACGGGAGGCAGCAG-3') and 806R (5'-GGACTACHVGGTWTCTAAT-3'). Amplicons were analyzed using the Bioanalyzer DNA 1000 (Agilent Technologies, USA). After filtering raw sequences, overlapping paired-end reads were merged with the tag, and the tag was clustered in a 97% sequence similarity OTU. The Greengenes reference database was used for bacterial classification with Mothur pipeline v. 1.39.5 as described previously [26]. Diversity indices were analyzed based on taxonomic ranks and OTUs. Metagenomic raw sequences were submitted to the Sequence Read Archive (SRA) with BioProject code SUB9919825 and BioSample accessions SRR14994872-SRR14994867.

### Statistical Analysis and Visualization of Results

Statistical differences between treatment means were performed through *t*-tests at *p* < 0.05 using SPSS (V25, IBM, USA). The built-in PCA function in Mothur was used to perform Principal Coordinate Analysis (PCoA) and visualized differences in treatments using bacterial community structure. Biomarker discovery of the bacterial community was determined using LEfSe (http://huttenhower.sph.harvard.edu/lefse/). Prediction of the functional profiles was conducted using the PICRUSt tool [27]. Briefly, the PICRUSt analysis conducted in this study employed a computational approach to predict functional profiles of soil metagenomes combining marker gene data and the reference genomes database. Using 16S information from OTUs extracted from the Mothur pipeline, the abundances of gene families were predicted in the different soil samples to provide useful insights into the potential functional attributes of uncultivated microbial communities found in NPK- and OC-treated jujube orchard soils. From the PICRUSt software package, gene content inference and metagenome inference were combined using the reference OTU tree and OTU tables, respectively, followed by gene content predictions and inferred metagenomes. The results were used to generate a gene table showing a functional gene count matrix. Specific functional genes were selected related to common soil enzyme, decomposition-related, and plant growth promotion-related enzyme-encoding genes. Statistically significant differences of these genes in different treatments were tested using Tukey’s test (SAS Institute, USA). Metagenome predictions used reference genomes and marker genes in the KEGG (Kyoto Encyclopedia of Genes and Genomes) database.

## Results

Organic amendments in agroecosystems can alter the chemical properties of soils. In this study, both the NPK-and OC-amended soils were acidic in nature (Table 1) with pHs ranging from 4.5-5.2. The OM content was 23.9~28.7 g/kg, showing no significant difference between organically and NPK-fertilized soils. The calcium and magnesium contents increased in OC-treated areas compared to the NPK-fertilized soil. Potassium (K), electric conductivity (EC) and NO_3_-N showed significant differences between NPK- and OC-amended soils.

Rarefaction curves indicated that the number of reads retrieved from the fertilized soils satisfactorily revealed the bacterial identities of the communities in those samples (Fig. S1). The numbers of OTUs were significantly greater in organic fertilizer-treated orchards than the NPK-treated soils at the 97% similarity level with more than 200 distinct OTUs detected (Table 2). The organic fertilizer-amended soils showed significantly greater Shannon and inv-Simpson indices than NPK-amended soils, and similar patterns were observed for richness indices (Chao and Ace).

**Fig. 1:**
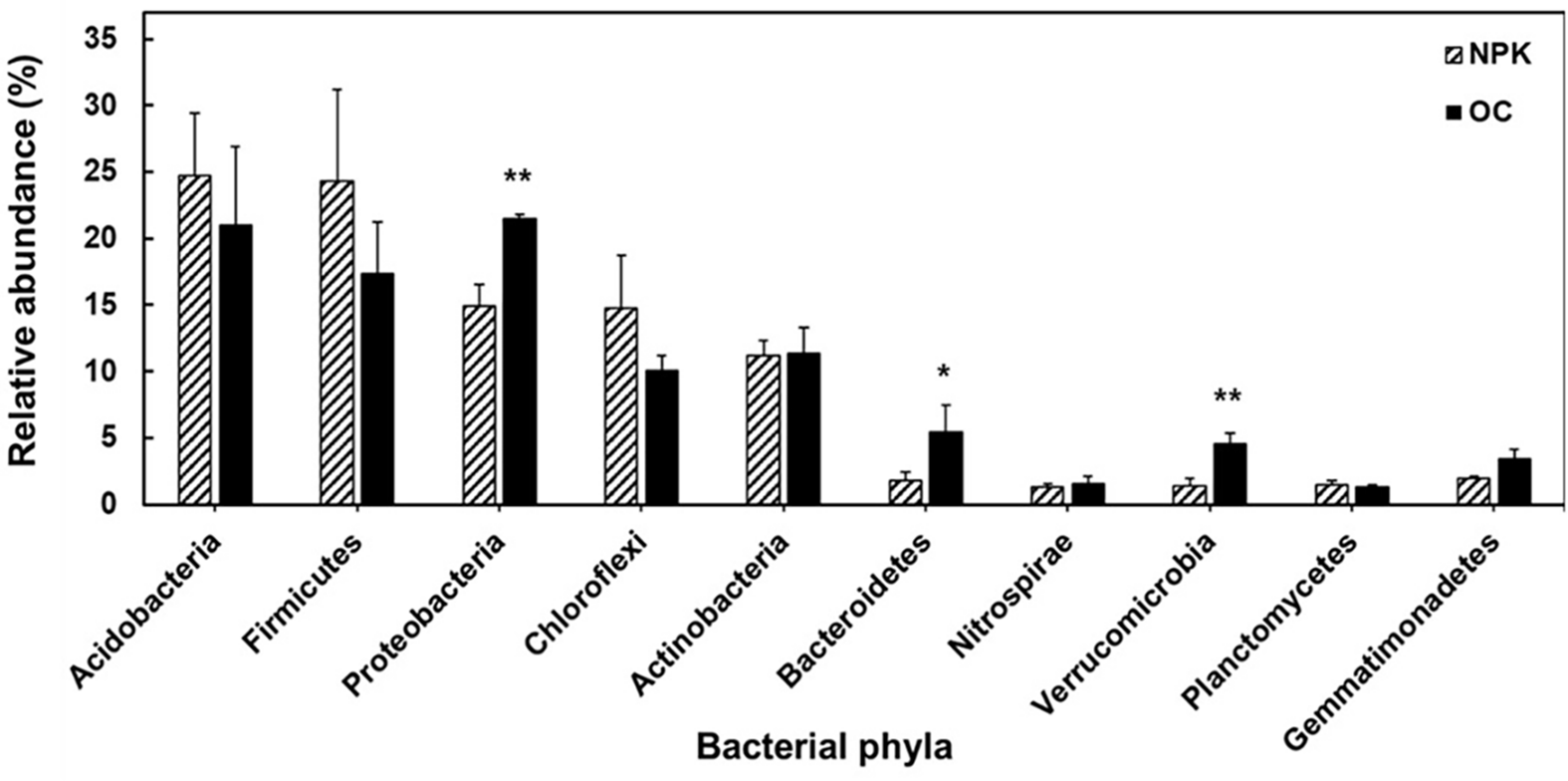
Top 10 most abundant phyla in fertilized soil samples. Significant differences are indicated by asterisks (*p < 0.05, **p < 0.01). NPK: jujube orchard soils treated with chemical fertilizers; OC: jujube orchard soils treated with organic fertilizer composed of oil cake.

**Fig. 2:**
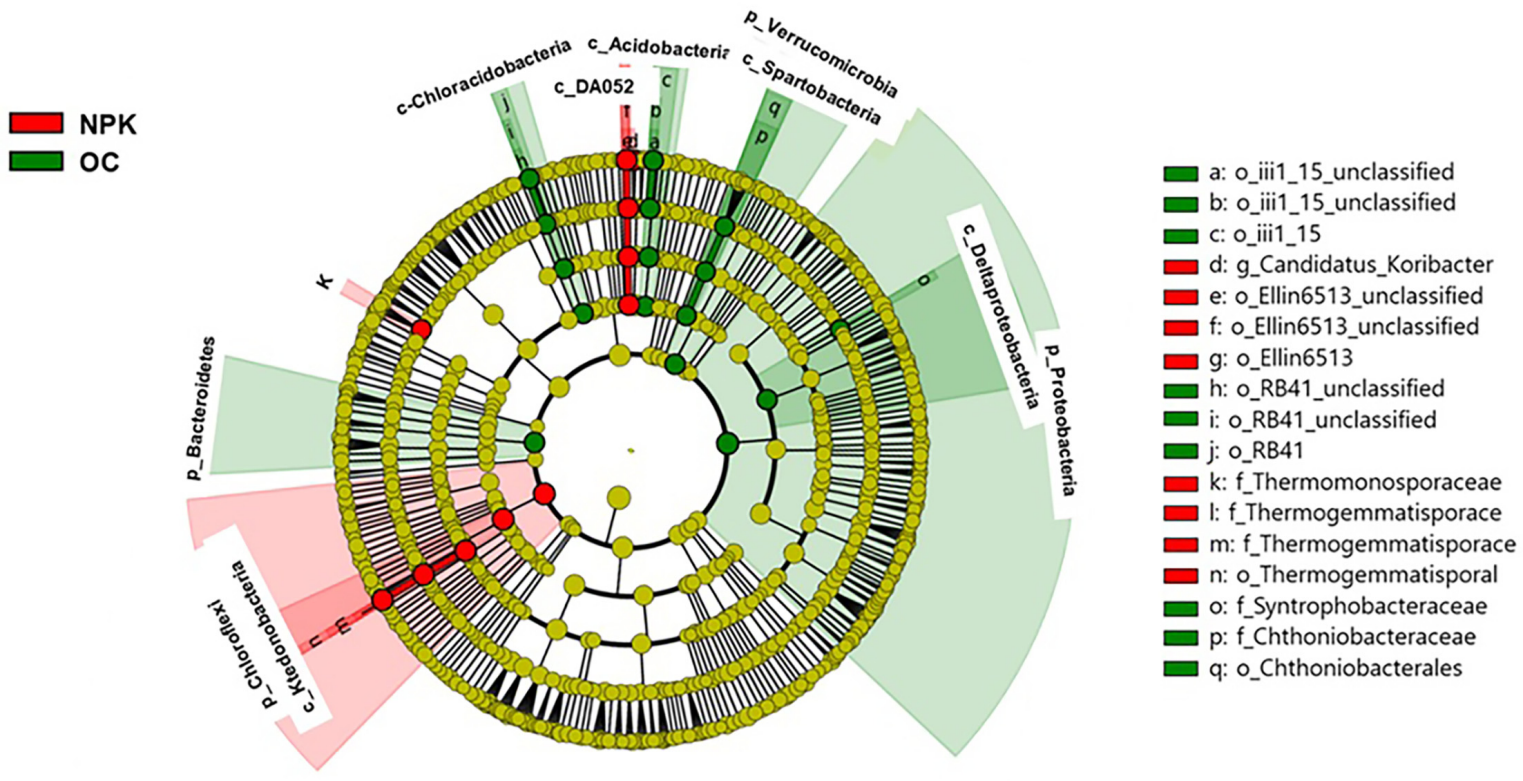
The LEfSe analysis showing significantly different bacterial groups comparing two treatments indicated by red and green dots. Bacterial taxa represented from the center outward from kingdom to genus. NPK: jujube orchard soils treated with chemical fertilizers; OC: jujube orchard soils treated with organic fertilizer composed of oil cake.

The bacterial community profile showed the soil samples to have 42 distinct and 34 common phyla. There were five dominant phyla with relative abundances of 10% or higher observed in both OC-fertilized and NPK-fertilized soils (Fig. 1). In comparing the distribution of bacteria at the phylum level, the soil treated with organic fertilizer was shown to possess Proteobacteria (21.5%), Acidobacteria (20.9%), Firmicutes (17.4%), Actinobacteria (11.4%), and Chloroflexi (10.1%) as its most dominant bacterial groups, whereas the NPK-fertilized soils were found to have Acidobacteria (24.7%), Firmicutes (24.3%), Proteobacteria (14.9%), Chloroflexi (14.8%), and Actinobacteria (11.2%) as its most abundant bacterial phyla. Proteobacteria, Bacteroidetes and Verrucomicrobia in OC-fertilized soil were significantly higher compared to NPK-fertilized soil. Furthermore, LEfSe showed the increase in abundances of Chloroflexi (Ktedonobacteria) and Acidobacteria in NPK-fertilized soil compared to OC-fertilized soils (Fig. 2), while the bacterial clades Deltaproteobacteria, Bacteroidetes, Chloracidobacteria, and Spartobacteria were more dominant in OC-amended soils. Details on the differences in OC- and NPK-fertilized soils at the bacterial order level are shown in Fig. S2.

**Fig. 3:**
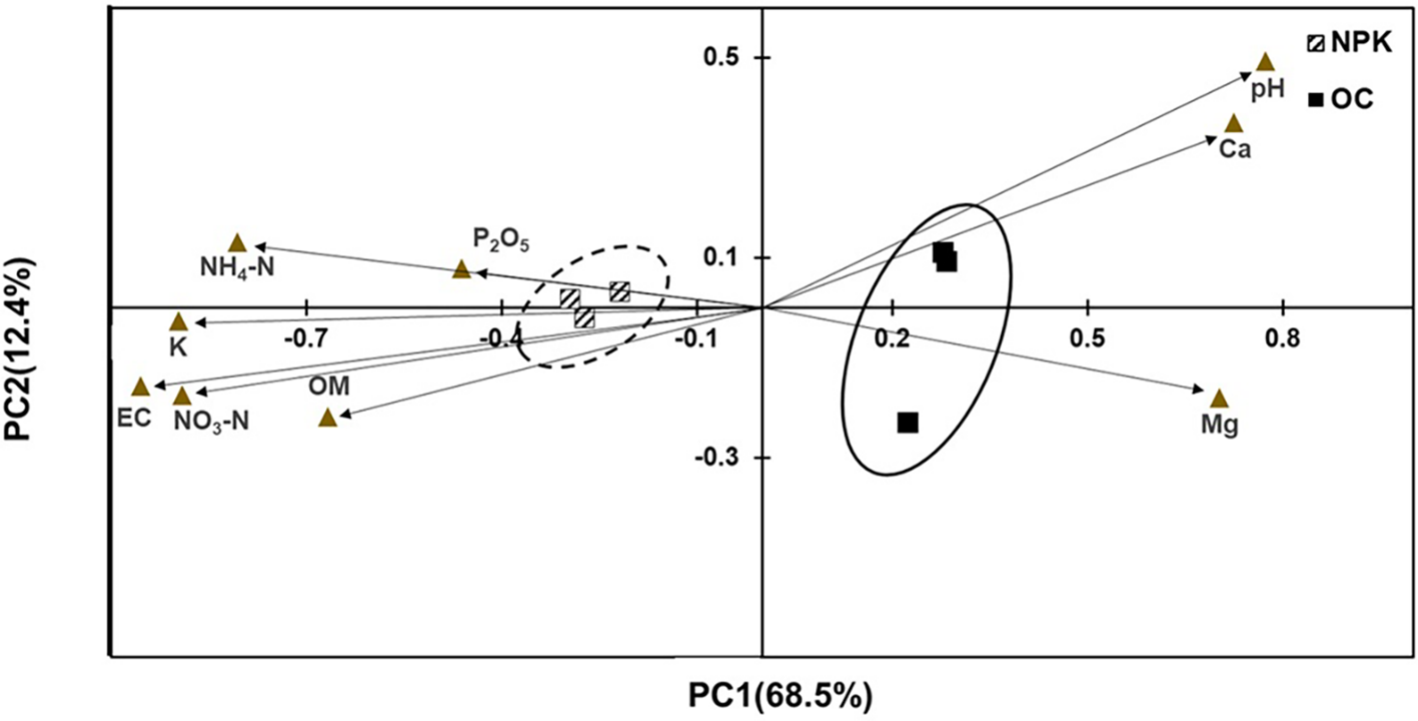
Grouping of the treatments determined by chemical properties of soils. NPK: jujube orchard soils treated with chemical fertilizers; OC: jujube orchard soils treated with organic fertilizer composed of oil cake.

The distinct community profiles of the studied soil groups are shaped by the chemical properties of the soil. The PCoA showed that soil pH, Ca, and Mg were bacterial community composition determinants in OC-amended soils, and the other parameters determined the community profiles of NPK-amended soils (Fig. 3). Distinct clustering of the treatment replicates dictated by soil chemical properties indicate clear variability between the OC- and NPK-treated jujube orchards. Principal component 1 (PC1) explained 68.5% of the overall variability in community structure while PC2 explained 12.4% with an overall cumulative variability of 80.9%, indicating considerable inclusion of main soil physicochemical factors explaining the overall variability observed. Furthermore, correlations of the microbial abundance and soil chemical parameters indicate a potential relationship between the abundances of specific microbial groups as affected by specific soil chemical parameters. Spearman correlation showed that the Verrucomicrobia population was positively correlated with soil pH and Ca content. Similarly, EC and NO_3_-N were positively correlated with Firmicutes, Chloroflexi, and Actinobacteria (Table 3). Additionally, Verrumicrobia and Nitrospirae showed a negative correlation with EC and NO_3_-N.

**Fig. 4:**
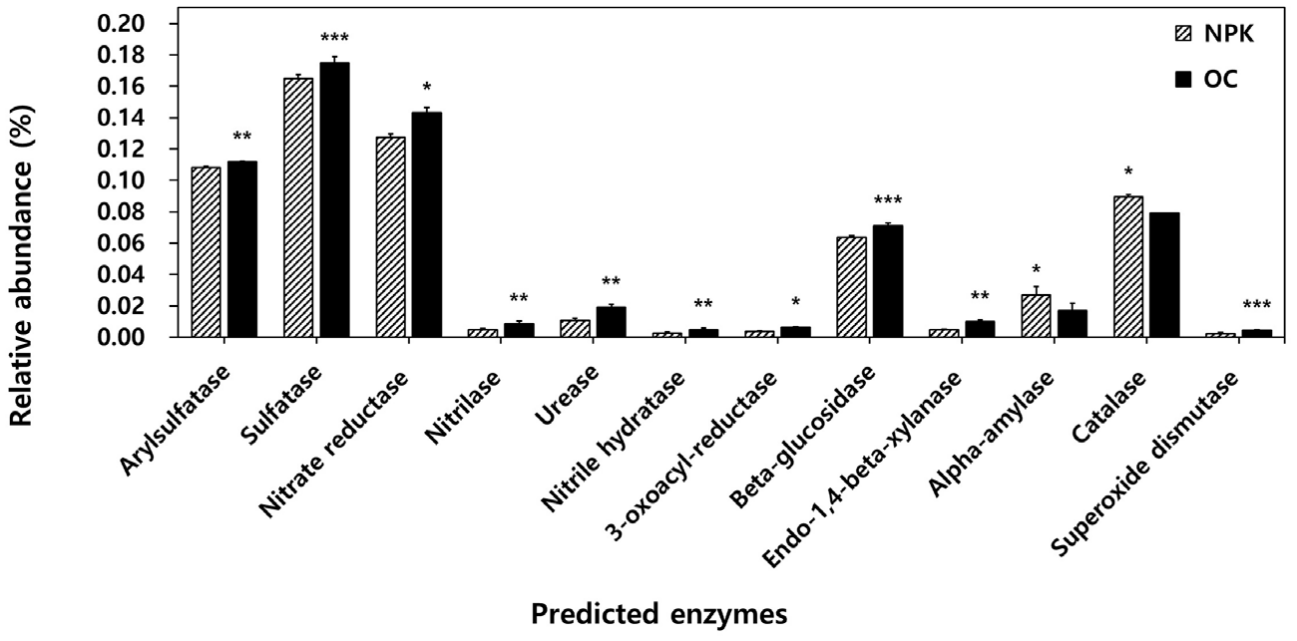
PICRUSt analysis predicting the relative abundance of common soil enzyme-encoding genes, decomposition-related enzyme-encoding genes, and plant growth promotion enzyme-encoding genes. Error bars indicated standard deviations. The asterisks mark the value of p: * at *p* ≤ 0.05, ** at *p* ≤ 0.01, *** at *p* ≤ 0.001. NPK: jujube orchard soils treated with chemical fertilizers; OC: jujube orchard soils treated with organic fertilizer composed of oil cake.

The functional profiles of the bacterial community were determined using PICRUSt. We divided the functional traits based on soil enzyme-encoding genes, decomposition-related genes, and plant growth promotion-related genes (Fig. 4). Among the typical soil enzyme-encoding genes, some of those detected were significantly higher in OC-amended soils. These include arylsulfatase, sulfatase, urease, nitrate reductase, nitrilase, nitrile hydratase, and 3-oxoacyl-reductase. Significantly abundant decomposition-related enzyme-encoding genes observed in OC-amended soils include beta-glucosidase and endo-1,4-beta-xylanase, while alpha-amylase is significantly abundant in the NPK-amended soils. Superoxide dismutase is significantly more abundant in the OC-amended soils related to plant growth promotion and stress responses while the NPK-treated soil has a significantly more abundant catalase enzyme-encoding gene.

## Discussion

In this study, we evaluated the impact of amending jujube orchards soils with an unconventional organic fertilizer made up of composted oil cake and compared the results with chemical fertilizer application. The effects of organic compost and NPK fertilization were evaluated with regards to the alterations in the soil chemical properties, the resulting changes in the bacterial community structure and diversity, and finally, the potential impact on predicted soil functions and activity.

### Impact of Oil Cake Amendment and Chemical Fertilization on Jujube Orchard Soil Chemistry

The application of organic fertilizers in agroecosystems can bring about significant alterations in the chemical and physical properties of soils [28, 29]. This is exemplified in the long-term, continuous application of either only organic fertilizer amendments, or mixed organic and inorganic chemical fertilizers, all of which change soil chemistry and ultimately affect functional and structural soil microbiomes [10, 11]. The most commonly used organic fertilizer amendments, at least in paddy fields, are fresh or composted animal manure, composted crop residues, such as rice straw, or a combination of both types [6]. The oil-cake organic fertilizer used in the study contained a combination of rapeseed cake, castor oil cake, and rice bran cake, and this was the source of nutrient input into the soils of jujube orchards. Oil cakes were observed to have higher nitrogen content compared to livestock compost [15] and they underwent rapid mineralization of organic nitrogen [12, 15], which potentially contributed to the nitrogen contents of the soil. The OM content showed no significant difference between organically and NPK-fertilized soils. Kim *et al*. [30] also suggested that the organic content of the oil cake fertilizer is more than 70% raw material, but it is easily decomposed due to the nature of the raw material and is not effective in increasing soil organic matter. In this study, both the NPK- and OC-amended soils were acidic. The soil pH affects soil nutrient availability directly correlated with productivity [31]. Calcium and magnesium contents increased in oil cake-treated areas compared to the NPK-fertilized soil. Since the calcium and magnesium content is low, it is estimated that the pH of the NPK-fertilized soil is more acidic than that of the OC-amended soil [32].

### Impact of Oil Cake-Based Organic Fertilizer and Chemical Fertilizer on the Bacterial Communities in Jujube Orchard Soils

Rarefaction curves showed that the reads from the fertilized soils satisfactorily reveal the bacterial identities of the communities. The present study also showed that OC-amended orchards have a significantly higher number of OTUs along with significantly higher diversity indices in contrast to the NPK-amended jujube orchards. This indicates that the application of organic fertilizer amendments resulted in increased members of other bacterial clades, magnifying the bacterial richness of OC-amended soils, and leading to the observed increase in diversity indices. The amendment of soils with organic fertilizer has been shown to enhance the population, diversity, and heterogeneity of microbial communities in soil [33, 34]. In a long-term organic amendment, the microbial populations of heterotrophic bacteria along with specific microbial guilds were increased, thereby altering the biological and chemical characteristics of the soils [35].

The application of different types of organic fertilizers, as well as different types of nitrogen sources and other kinds of fertilizer amendments, impact the microbial guilds differently. For instance, the application of cattle versus swine manure has led to enrichment of methanogens belonging to *Methanomicrobiaceae* not observed in soils under swine manure application [36]. In addition, there are clade-specific changes related to nitrogen cycling microbial communities as influenced by nitrogen fertilization [37]. The differing responses of diverse microbial guilds may show common trends as well as taxon-specific or clade-specific microbial responses to different types of fertilization input in agricultural lands which could be better elucidated under long-term fertilization studies [6, 10, 11].

In terms of the differences in bacterial clades between OC- and NPK-amended soils, clear significant differences occur in the dominance of bacterial taxa from phylum to class and lower taxonomic groups. At the phylum level, Proteobacteria, Bacteroidetes, and Verrucomicrobia in organically fertilized soil were significantly higher compared to NPK-fertilized soil. These trends are also observed in other studies showing that inorganic fertilization regimes decrease the population of Proteobacteria and Verrucomicrobia [38, 39]. Additionally, Proteobacteria contains bacterial populations that are important for carbon, nitrogen, and sulfur cycling in soil [40]. Proteobacteria is also the most dominant bacterial group where most plant growth-promoting bacteria belong mainly to different crop plants [41-44]. Bacteroidetes contain several polysaccharide-degrading gene-encoding enzymes [45, 46], which are believed to play an important role in lignocellulosic degradation present in high abundance in organically fertilized soils [47]. Furthermore, LEfSe showed an increase in abundances of Chloroflexi and Acidobacteria in NPK-fertilized compared to OC-fertilized soils. This was similarly observed in a previous study showing increased abundances of Acidobacteria in soil amended with chemical fertilizers [48]. On the other hand, Proteobacteria were abundant in organic fertilizer-treated soil, which was consistent with a previous study where sugarcane fields amended with organic fertilizer had also shown higher abundances of Proteobacteria [49].

Principal component analysis showed that the distinct community profiles of the studied groups were mainly attributed to the soil chemical properties. The OC-amended soils were mainly affected by soil pH, Ca, and Mg, while the variabilities of NPK-amended soils were determined by other soil parameters. These observations are supported by positive and negative correlations of specific soil chemical parameters and different bacterial populations found in each treatment. A previous study supports our observations, where nitrogen treatment augmented the ratio of Actinobacteria and Firmicutes and diminished the ratio of Acidobacteria and Verrucomicrobia in soils [50]. The differential impact of fertilizer application on soil chemical parameters also leads to changes in the community structure, diversity, and abundance of microbial groups in agricultural lands. As observed in our study, the clear clustering of the OC- and NPK-amended soils mainly influenced by soil chemical parameter and different microbial guilds indicates significant variabilities that exist between the two jujube orchard soils treated with different fertilizer regimes. These differences in terms of soils become prominent, particularly in long-term application of compost, NPK, and compost-plus NPK treatments compared to the control creating characteristic soils with different physicochemical properties and varying microbial community composition, diversity, and abundances, which eventually lead to changes in functional activities occurring in the soils [6, 10, 11].

### Impact of Oil Cake-Based Organic Fertilizer and Chemical Fertilizer on Microbial Functional Diversity of Jujube Orchard Soils

The functional profiles mainly based on the community composition and abundance also show significant differences between OC- and NPK-amended soils. Arylsulfatase, sulfatase, urease, nitrate reductase, and nitrilase are involved in the sulfur and nitrogen cycle, and 3-oxoacyl-reductase is metabolically related to the biosynthesis of fatty acids and polyunsaturated fatty acids. Arylsulphatase hydrolyzes soil sulfate esters [51, 52], while sulfatase is induced by sulfate restriction and affects the plant growth promotion of some bacteria [51]. Soil sulfatase activity indicates soil health after fertilizations as it reflects soil microbial activity [53]. The higher urease activity in OC-treated soils was similarly observed in farmyard manure-treated paddy fields and this increase led to the production of the end product, ammonium ions [35]. All these enzymes can be typically attributed to various plant-based organic fertilization amendments in agricultural soils.

Organic decomposition-related functional genes encoding beta-glucosidase and endo-1,4-beta-xylanase were significantly higher in organically fertilized soil. Beta-glucosidase hydrolyzes various beta-glucosides present in plant debris [54]. Increase in beta-glucosidase was also observed in long-term organically fertilized paddy fields using composted farmyard manure. Endo-β-1,4-xylanase hydrolyzes β-1,4 bonds in plant xylan found in hemicellulose [55]. Also, superoxide dismutase among plant growth-promoting enzymes was high in OC-amended soil. Superoxide dismutase is important in catalyzing superoxide anions to hydrogen peroxide (H_2_O_2_) and O_2_ and protecting against oxidative stress [56]. It is thought that superoxide dismutase can confer plant and microbial resistance to environmental stresses.

In general, the changes in the functional profile or microbial functional diversity observed in different soils are due to the combination of alterations in the chemical properties of the soil concurrent to the shifts in bacterial community structure, diversity, and abundance. The functions provided by soils are mainly dependent on microbial actions attributed to particular microbial clades capable of these functions [8, 9]. For example, methane emission dictated by methane production and methane and methanol oxidation is dependent on methanogenic archaea and methanotrophic and methylotrophic bacterial communities [6, 10]. This is also true for nitrogen transformative processes in agricultural soils where many processes are linked to the action of nitrogen cycling-related microbial guilds [11].

Our study supports that the application of organic fertilizer in the form of oil cake into jujube orchards enhances the soil quality, leading to increased richness and diversity of bacterial communities. It would be interesting to investigate whether long-term input of oil cake amendments can lead to similar observations in long-term organic fertilized lands with increased nutrient content, to include soil labile carbon and nitrogen pools that enhance soil enzymatic activities as well as microbial populations and diversity [10, 11, 35].

## Supplemental Materials

Supplementary data for this paper are available on-line only at http://jmb.or.kr.



## Figures and Tables

**Table 1:** Chemical properties of soils amended with chemical fertilizer (NPK) and organic fertilizer mainly composed of oil cake (OC).

Treatment	pH	OM	P_2_O_5_	K	Ca	Mg	EC	NO_3_-N	NH_4_-N
	(1:5)	g kg^-1^	mg kg^-1^		cmol^+^ kg^-1^		dS m^-1^		mg kg^-1^
NPK	4.5 ± 0.4	28.7 ± 4.9	510.0 ± 175.0	2.23 ± 0.46*	3.6 ± 0.8	2.2 ± 0.5	3.12 ± 0.55*	187.4 ± 55.4*	67.5 ± 22.2
OC	5.2 ± 0.4	23.9 ± 1.4	365.3 ± 91.6	1.19 ± 0.14	4.8 ± 0.8	2.7 ± 0.1	1.14 ± 0.35	13.2 ± 4.7	5.0 ± 2.8

^♩^Mean separation by *t*-test at *p < .05, **p < .01

**Table 2:** Calculated α-diversity indices and summary of sequences and OTUs derived from 16S rRNA sequencing.

Treatment	No. of sequences	Goods coverage estimator (%)	No. of OTUs	Chao	Ace	Shannon index	Inv-simpson index
NPK	15841	98.5	1151.2 ± 93.4	1323.1 ± 118.7	1314.4 ± 114.9	5.7 ± 0.1	100.9 ± 26.5
OC	15841	98.4	1372.1 ± 32.6*	1524.6 ± 42.4*	1516.0 ± 42.7*	6.2 ± 0.1*	181.7 ± 24.3*

^†^OC : organic fertilizer composed of oil cake, OTU : operational taxonomic units

^♩^Mean separation by *t*-test at *p < .05

**Table 3:** Spearman’s rho values (R) representing the significant correlation between the bacterial phyla and soil chemical properties.

Bacterial phyla	Soil chemical property					
	pH	Ca	K	EC	NO_3_-N	NH_4_-H
Firmicutes	-0.828**	-0.762*	0.600	0.817**	0.817**	0.333
Proteobacteria	0.418	0.402	-0.917**	-0.517	-0.517	-0.717*
Chloroflexi	-0.820**	-0.854**	0.467	0.833**	0.833**	0.267
Actinobacteria	-0.728*	-0.661	-0.083	0.683*	0.683*	-0.300
Bacteroidetes	0.427	0.444	-0.717*	-0.633	-0.633	-0.417
Verrucomicrobia	0.803**	0.820**	-0.550	-0.783*	-0.783*	-0.283
Gemmatimonadetes	0.510	0.410	-0.850**	-0.600	-0.600	-0.850**
Bacteria_unclassified	0.753*	0.753*	-0.533	-0.733*	-0.733*	-0.367
Nitrospirae	0.887**	0.862**	-0.217	-0.817**	-0.817**	0.017
