# Supplementary material for: Shifts in Soil Bacterial Community Composition of Jujube Orchard Influenced by Organic Fertilizer Amendment
Source: J Microbiol Biotechnol. 2024 Oct 30;34(12):2539–46. doi: 10.4014/jmb.2406.06037 (PMC11729348; doi:10.4014/jmb.2406.06037)
Supplement: Supplementary file 1 [file jmb-34-12-2539-supple.pdf]

J. Microbiol. Biotechnol. 2024. 34(12): 2539–2546
https://doi.org/10.4014/jmb.2406.06037

Supplementary Figures

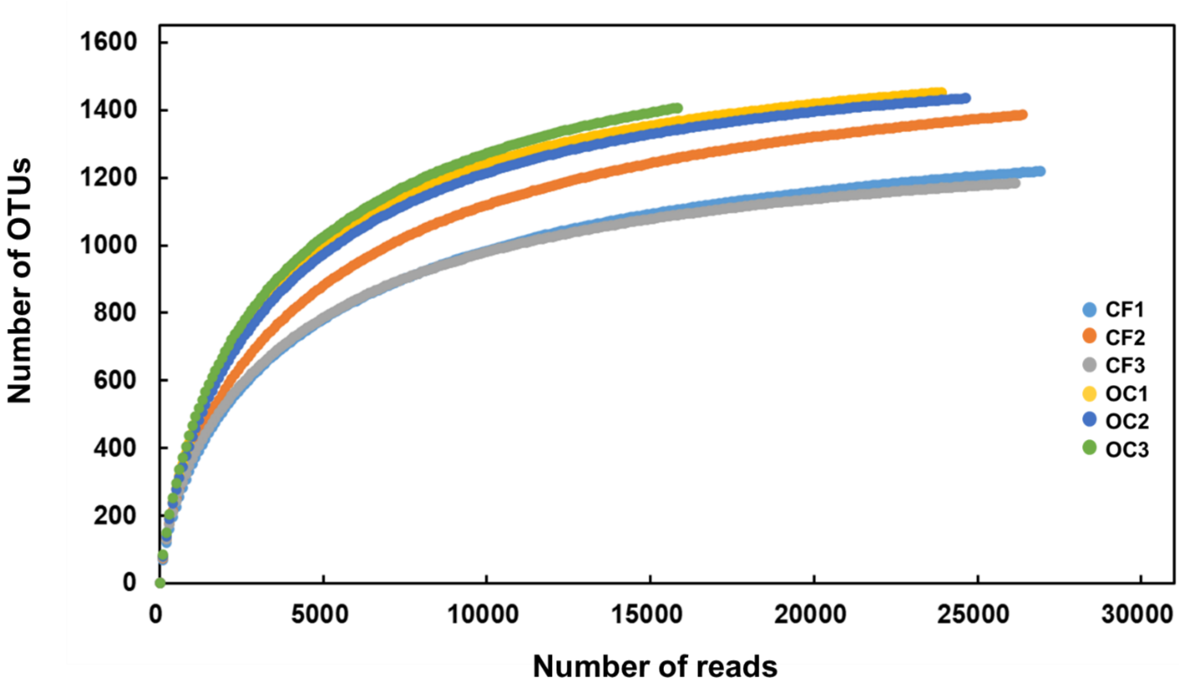

Fig. S1. The rarefaction curve of bacterial 16SrRNA sequences obtained from the studied soil groups.

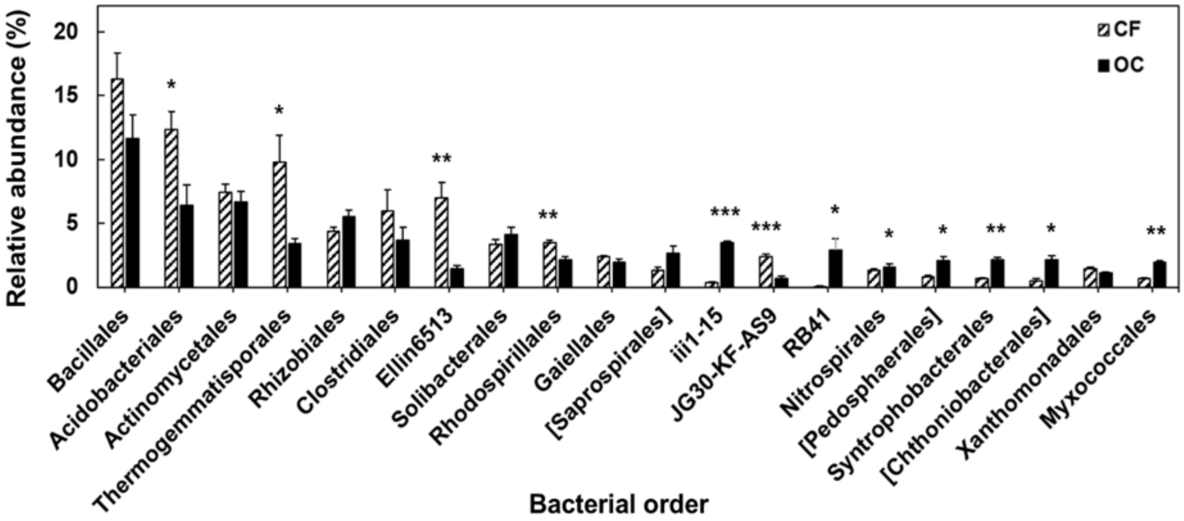
CF
OC
Relative abundance (%)
20
15
10
5
0
*
*
**
**
***
***
*
*
*
**
*
**
Bacillales
Acidobacteriales
Actinomycetales
Thermogemmatisporales
Rhizobiales
Clostridiales
Ellin6513
Solibacterales
Rhodospirillales
Gaiellales
[Saprospirales]
iii1-15
JG30-KF-AS9
RB41
Nitrospirales
[Pedosphaerales]
Syntrophobacterales
[Chthoniobacterales]
Xanthomonadales
Myxococcales
Bacterial order

Fig. S2
